# Abdominal Schwannoma Mimicking Lymph Node Metastasis in Rectal Cancer

**DOI:** 10.7759/cureus.33096

**Published:** 2022-12-29

**Authors:** Enxhi Kotrri, Derek Jonker, Rebecca Auer, Gordon Locke

**Affiliations:** 1 Faculty of Medicine, University of Ottawa, Ottawa, CAN; 2 Division of Medical Oncology, University of Ottawa, The Ottawa Hospital, Ottawa, CAN; 3 Division of General Surgery, University of Ottawa, The Ottawa Hospital, Ottawa, CAN; 4 Division of Radiation Oncology, University of Ottawa, The Ottawa Hospital, Ottawa, CAN

**Keywords:** lymph node metastasis, radiation oncology, lymph node (ln), metastasis, rectal cancer, abdominal schwannoma, schwannoma

## Abstract

Schwannomas are rare benign tumors that are often asymptomatic and identified incidentally on imaging studies undertaken for another purpose. Schwannomas arising from the vestibular nerve are the most common site of identification; however, schwannomas can arise extracranially in any peripheral nerve tissue. Here, we present a case study of a patient with a localized rectal adenocarcinoma who was found to have a retroperitoneal schwannoma initially felt to be a lymph node metastasis of his rectal cancer. The diagnosis of schwannoma was confirmed via biopsy, which resulted in changes to the patient’s overall management including radiotherapy volumes and recommendation against neoadjuvant or adjuvant systemic therapy.

## Introduction

Rectal cancers are predominantly adenocarcinoma (>90%), and patients commonly present with a change in bowel habits, abdominal pain, and/or constitutional symptoms [1]. The treatment of rectal cancer varies depending on the stage of the disease but generally includes a combination of radiotherapy, chemotherapy, and surgical excision [2]. Early-stage rectal cancers can be managed with total mesorectal excision with low rates of local or distant recurrence. When there is invasion through the muscularis propria of the rectum, there are higher rates of local recurrence with surgery alone. Radiotherapy can be used neoadjuvantly or adjuvantly to reduce the risk of local recurrence. When there is evidence of lymph node metastases, chemotherapy is typically recommended to reduce the risk of recurrence and may be given prior to surgical resection.

Schwannomas are rare benign tumors that originate from Schwann cells of peripheral nerve sheaths. Schwannomas are associated with the cranial nerves and most commonly arise from the vestibular portion of the eighth cranial nerve, known as vestibular schwannoma, which accounts for approximately 8% of intracranial tumors in adults [3]. Given their origin, however, schwannomas can develop anywhere in the body where nerves reside and hence may also be found extracranially [3]. About 25%-45% of extracranial schwannomas reside in the head and neck region due to the abundance of nerves in this region but are also commonly found in the extremities and trunk [4]. More rarely, however, they have been identified in uncommon areas such as the abdominal wall, multiple intramuscular regions, retroperitoneal space, intrathoracic space, and cutaneous areas [5,6]. Although schwannomas can present with a wide range of symptoms depending on location and impact on surrounding nerves, they typically tend to be asymptomatic due to their benign properties. Therefore, they can pose a diagnostic dilemma when identified incidentally, and histopathology remains the gold standard for diagnosis [7]. In this case report, we present a case of a schwannoma identified incidentally mimicking lymph node metastasis in a patient with rectal cancer.

## Case presentation

We present a case report of a 69-year-old male with a past medical history of dyslipidemia, aortic regurgitation, cerebral hemorrhage (2018), and focal epilepsy secondary to the aforementioned hemorrhage. His past surgical history was significant for inguinal hernia repair, appendectomy, and tonsillectomy. He also had an oncologic history of thymoma in 2011 for which he underwent surgical resection and adjuvant radiotherapy in 2012.

**Figure 1:**
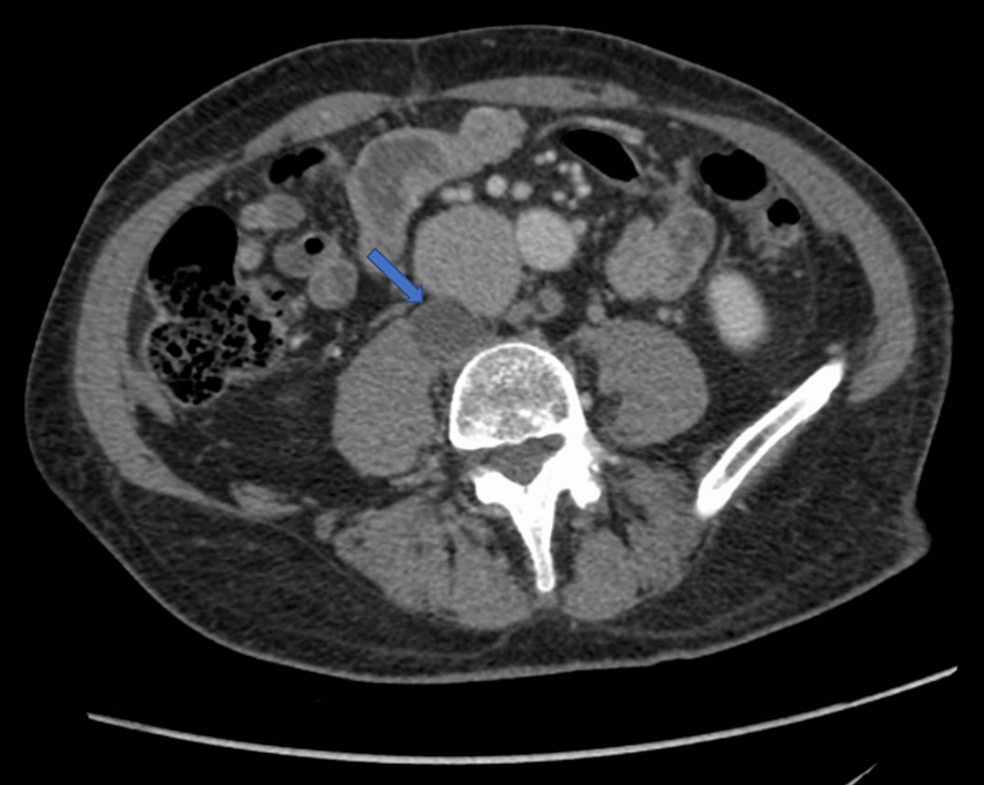
CT of the abdomen and pelvis with IV contrast The following CT scan image demonstrates an enlarged necrotic-appearing solitary nodule in the retrocaval region, posterior to the inferior vena cava and slightly to the right of the midline in the lower lumbar spine region (arrow). The nodule occupies the location of a lymph node and measures 28 × 24 mm. CT: computed tomography, IV: intravenous

He presented with a change in bowel habits and rectal bleeding in the fall of 2021, during which time he was also found to have a positive fecal immunochemical test (FIT). He underwent a colonoscopy in December 2021 for further follow-up, which demonstrated a 4 × 3 cm rectal adenocarcinoma located posteriorly 8 cm from the anal verge. Subsequently, he underwent computed tomography (CT) scan as part of staging investigations, which showed the rectal adenocarcinoma as a lobulated structure in the mid-rectum but most significantly also identified a necrotic 2.4-cm retroperitoneal lymph node in the retrocaval region at the level of L5, suspicious for metastatic disease (Figure 1). His carcinoembryonic antigen (CEA) level was within normal limits at 3.3 ug/L.

**Figure 2:**
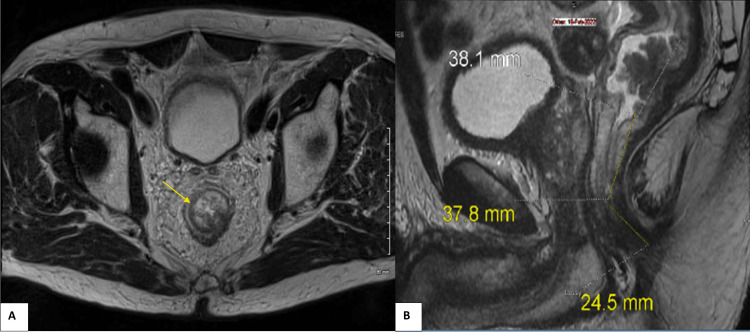
MRI of the pelvis without contrast undertaken for local rectal cancer staging The following T2-weighted MRI images demonstrate a polypoid mass (A) (arrow) in the mid-rectum with a craniocaudal length of about 3.8 cm (B). MRI: magnetic resonance imaging

The patient then underwent magnetic resonance imaging (MRI) of his pelvis, which showed a noncircumferential polypoid rectal mass with a base diameter of 39 mm arising from the posterior wall of the mid-rectum 8 cm from the anal verge (Figure 2). There was a maximum extramural depth of invasion of 3 mm but a clear mesorectal fascia (MRF) and no extramural vascular invasion (EMVI). There was no adenopathy in the mesorectum; however, there was a large solitary retroperitoneal mass initially thought to be a necrotic lymph node, and therefore, his rectal cancer was staged as a cT3bN0M1, as the retroperitoneal lymph nodes are outside of the regional lymphatic territory of mid-rectal tumors.

**Figure 3:**
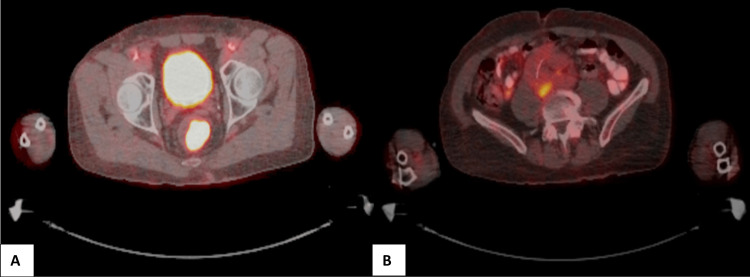
PET scan images The following PET scan images demonstrate the intensely FDG avid lesion in the mid-rectum corresponding to the known primary (A) and the low-density retrocaval mass suspicious for a metastatic lymph node (B). PET: positron emission tomography, FDG: fluorodeoxyglucose

Given the unusual appearance of the presumed lymph node metastasis, the patient subsequently had a positron emission tomography (PET) scan to determine if this was malignant. The PET scan showed an intensely fluorodeoxyglucose (FDG) avid lesion in the mid-rectum with a maximum standardized uptake value (SUV) of 25.33 corresponding to the known rectal primary (Figure 3A). There was also a low-density retrocaval enlarged mass measuring 27 × 23 mm demonstrating a mildly increased maximum SUV of 4.02 (Figure 3B). The mild uptake was reported to likely be an underestimate due to the central necrotic nature of the node, and the abnormal findings were most consistent with metastatic node involvement. No other enlarged or active lymph nodes were found in the rest of the retroperitoneum, mesentery, or pelvic/inguinal stations. There was no evidence of distant metastatic disease on the remainder of the PET scan.

The patient had laboratory investigations to rule out pheochromocytoma and paraganglioma, which were also on the differential. The results came back within the normal range (Table 1).

**Table 1:** Laboratory investigations Laboratory values were within normal limits, ruling out pheochromocytoma and paraganglioma.

	Reference range	Result
Creatinine urine	Latest reference range: 3.5-24.5 mmol/L	5.9 mmol/L
Creatinine per day	Latest reference range: 7.8-20 mmol/day	8 mmol/day
Total volume (24-hour urine)	Latest units: L/day	1.35 L/day
Free 3-methoxytyramine (24-hour urine)	Latest reference range: <0.52 umol/day	0.12 umol/day
Free 3-methoxytyramine	Latest units: umol/L	0.09 umol/L
Free metanephrine	Latest units: umol/L	0.08 umol/L
Free metanephrine (24-hour urine)	Latest reference range: <0.25 umol/day	0.11 umol/day
Free normetanephrine	Latest units: umol/L	0.06 umol/L
Free normetanephrine (24-hour urine)	Latest reference range: <0.28 umol/day	0.08 umol/day

As management would be significantly changed by lymph node involvement, a multidisciplinary cancer conference recommended a CT-guided core biopsy. The suspicious retroperitoneal lymph node was sampled with a core needle biopsy, and the pathology report revealed a spindle cell neoplasm with cellular fibrillary areas and paucicellular microcystic areas, but no apparent nuclear atypia or mitoses. Immunohistochemistry showed strong immunoreactivity of the neoplastic cells for protein S100 but negative for the following proteins: smooth muscle actin (SMA), cluster of differentiation 34 (CD34), AE1/AE3, and cytokeratin 8 (CK8)/CK18. The pathological features were consistent with a schwannoma, which reclassified the patient as a clinical cT3BN0 mid-rectal cancer with a clear circumferential resection margin (CRM) and no EMVI.

As the patient was node-negative with no high-risk features, neoadjuvant chemotherapy was not recommended. He underwent neoadjuvant radiotherapy to a dose of 25 Gray (Gy) in five fractions over the course of a week. Following the completion of neoadjuvant radiotherapy, he had a repeat MRI of the pelvis, which showed that the tumor reduced in size (1.7 cm from 3.8 cm pretreatment) and wall thickness (2 cm from 3.2 cm pretreatment). He then underwent definitive surgical management 12 weeks following the completion of radiotherapy, which consisted of an ultra-low anterior resection with coloanal anastomosis. The patient tolerated the surgery well with no complications and good postoperative recovery. The patient’s final pathology demonstrated a ypT2N0 moderately differentiated adenocarcinoma with 0/16 lymph nodes involved, and no lymphovascular or perineural invasion resected with negative margins. He was assessed postoperatively, and no adjuvant systemic therapy was recommended.

Following his treatment, the patient remained systemically well. He continued to have episodes of hematochezia with defecation following his radiation, but otherwise, his bowel habits returned to near pretreatment baseline, and he is now undergoing oncologic surveillance for his rectal cancer with no evidence of recurrent disease at the time of his last follow-up. He is also proceeding with surveillance for his schwannoma at this time, which has been stable.

## Discussion

Schwannomas are rare benign tumors that arise from Schwann cells of the peripheral nerve and are mainly located in the peripheral nerves or central nervous system, although they can theoretically be found anywhere in the body where nerves reside [8]. Typically, schwannomas occur unilaterally as solitary encapsulated tumors; however, patients with neurofibromatosis are at increased risk of schwannoma and may present with these tumors in more than one site [5,9]. Schwannomas can cause a variety of symptoms depending on their specific location in the body and the compressive effects of surrounding structures, although they commonly tend to be asymptomatic due to their slow-growing and benign properties [6]. They are especially rare in the retroperitoneal cavity, accounting for only 0.7%-2.7% of schwannomas, where they are associated with a lack of symptomatology [6]. Given their benign nature, schwannomas in the retroperitoneum (as with many other locations) are usually incidental radiographic findings in the process of investigating another unrelated etiology. There are multiple cases in the literature in which retroperitoneal schwannomas have been identified incidentally mimicking other tumors such as hepatic tumors, adrenal tumors, and ovarian neoplasm in women [10-13]. Similar to this patient’s case, there are also cases of retroperitoneal schwannomas mimicking metastatic diseases, such as one case of a schwannoma mimicking necrotic lymph node metastasis from bladder cancer and another case of a schwannoma mimicking lymph node metastasis of seminoma [14,15]. Therefore, the initial identification of these lesions can often lead to diagnostic uncertainty, and the definitive diagnostic standard for schwannomas remains biopsy for anatomopathological assessment [8].

In this case, a retroperitoneal schwannoma was initially identified on CT imaging in the process of staging a rectal adenocarcinoma, thereby mimicking retroperitoneal lymph node metastasis of rectal carcinoma and posing diagnostic uncertainty. Further investigations were undertaken to identify the etiology of the lesion as the management of rectal cancers varies considerably depending on the stage of the disease. The initial recommended management when the lesion identified was thought to be evident of metastatic lymph node involvement was total neoadjuvant therapy consisting of short-course radiotherapy of 25 Gy in five fractions, followed by six cycles of capecitabine and oxaliplatin (CAPOX) chemotherapy (capecitabine 1,000 mg/m^2^ orally twice daily on days 1-14, oxaliplatin 130 mg/m^2^ intravenously on day 1, and a chemotherapy-free interval between days 15-21), followed by definitive surgical excision of the tumor shortly thereafter as per the Rectal cancer And Preoperative Induction therapy followed by Dedicated Operation (RAPIDO) trial [16]. However, it should be noted that while practices for treating stage III rectal cancer vary between centers as some have yet to adopt the RAPIDO approach, the treatment of stage III rectal cancer always includes chemotherapy.

Once the biopsy results returned positive for schwannoma, this ruled out lymph node metastasis and reclassified the patient as cT3BN0, a more favorable stage of the disease. Chemotherapy was no longer recommended; therefore, he underwent a different treatment course for early-stage rectal cancer consisting of a short course of radiotherapy (25 Gy in five fractions), followed by surgical resection 12 weeks later. By pursuing a biopsy and correctly diagnosing this schwannoma, the patient avoided overtreatment and the toxicities associated with chemotherapy. More specifically, in avoiding CAPOX chemotherapy, he avoided the >80% risk of neuropathy, which can be grade III (intolerable paresthesias affecting function) in 4% and even permanent in some patients [17]. He also avoided the approximately 1% risk of death from chemotherapy due to complications such as febrile neutropenia and severe diarrhea.

## Conclusions

Schwannoma is a rare benign tumor that is often asymptomatic and is identified incidentally on imaging studies undertaken for another reason. It often poses diagnostic uncertainty when identified as it can mimic another etiology and the standard of diagnosis remains histopathologic via biopsy. Overall, this case highlights how the incidental discovery of a schwannoma that was mimicking lymph node metastasis changed the patient’s management plan after being identified on biopsy, thereby allowing him to receive the most optimal treatment for his stage of disease.
